# Transcriptomic analysis of the regulation of stalk development in flowering Chinese cabbage (*Brassica campestris*) by RNA sequencing

**DOI:** 10.1038/s41598-017-15699-6

**Published:** 2017-11-14

**Authors:** Xinmin Huang, Yuling Lei, Hongling Guan, Yanwei Hao, Houcheng Liu, Guangwen Sun, Riyuan Chen, Shiwei Song

**Affiliations:** Guangdong Provincial Engineering Technology Research Center for Protected Horticulture, College of Horticulture, South China Agricultural University, Guangzhou, 510642 Guangdong, China

## Abstract

Flowering Chinese cabbage is a stalk vegetable whose quality and yield are directly related to stalk development. However, no comprehensive investigations on stalk development have been performed. To address this issue, the present study used RNA sequencing to investigate transcriptional regulation at three key stages (seedling, bolting, and flowering) of stalk development in flowering Chinese cabbage. Anatomical analysis revealed that cell division was the main mode of stalk thickening and elongation at all key stages. Among the 35,327 genes expressed in shoot apices, 34,448 were annotated and 879 were identified as novel transcripts. We identified 11,514 differentially expressed genes (DEGs) among the three stages of stalk development. Functional analysis revealed that these DEGs were significantly enriched in ‘ribosome’ and ‘plant hormone signal transduction’ pathways and were involved in hormone signal transduction, cell cycle progression, and the regulation of flowering time. The roles of these genes in stalk development were explored, and a putative gene-regulation network for the stalk flowering time was established. These findings provide insight into the molecular mechanisms of stalk development in flowering Chinese cabbage that provides a new theoretical basis for stalk vegetable breeding.

## Introduction

*Brassica* spp. vegetables belong to the Brassicaceae (Cruciferae) family and include different types of cabbage (green, red, etc.), cauliflower, broccoli, Brussels sprouts, and kale, all of which are grown and consumed worldwide^1,2^. *Brassica* species exhibit extensive diversity, with a wide range of morphological and phytochemical characteristics. In addition, related cultivars accumulate secondary metabolites that have beneficial effects on human health, including glucosinolates and phenolics, which are anti-carcinogenic and antioxidant compounds^1,3,4^. Leafy heads and enlarged organs (roots, stems, and inflorescences) are important morphological characteristics in terms of the economic value of *Brassica* spp. crops^5,6^. Many studies have been performed to examine the development of leafy heads in Chinese cabbage (*B*. *rapa*) and cabbage (*B*. *oleracea*), as well as the roots or stem tubers in turnip (*B*. *rapa* subsp. *rapa*), kohlrabi (*B*. *oleracea*), swede (*B*. *napus*), and tuberous mustard (*B*. *juncea*)^7,8^, but little information in available regarding stalk development in *Brassica* spp., such as flowering Chinese cabbage (*B*. *campestris*), Chinese kale (*B*. *alboglabra*), and purple cai-tai (*B*. *campestris*).

Stalk *Brassica* vegetables develop through a bolting process that includes stem elongation, thickening, and flowering. Plant hormones such as gibberellins (GAs), auxin, cytokinin (CTK), and brassinolide (BR) regulate stalk development through complex signal transduction^9^. *In vitro*, GA3 promotes stem elongation and bolting in spring rape (*B*. *napus*), and blocking GA biosynthesis with chlorocholine chloride suppresses stem elongation^10^. A reduction in indole-3-acetic acid (IAA), CTK, and BR contents or partial blockage of signal transduction were previously found to inhibit stem elongation^11–14^. Exogenous application of abscisic acid (ABA) stimulated tuber thickening in potato^15^, and ABA signalling was implicated in stem inflation in tuberous mustard^7^. In parallel, these hormones interact with each other and form a complex regulatory network^9^. In addition, these plant hormones affect genes associated with cell cycle regulation, such as those encoding cyclin-dependent kinases (CDKs) and cyclins (CYCs) that affect stalk development^16–18^.

Flowering is an important aspect of stalk development and represents an important transition from the vegetative to the reproductive stage^19^. In the model plant *A*. *thaliana*, >200 genes controlling flowering time have been identified^19,20^ that form a complex network of six pathways regulating photoperiod, autonomy, vernalization, GA signalling, response to ambient temperature, and age^19–21^. *Brassica* spp. are closely related to *A*. *thaliana* and many flowering time-related gene orthologues have been identified in these vegetables^22–24^, including Chinese cabbage (http://brassicadb.org/brad/flowerGene.php#).

Flowering Chinese cabbage (*B*. *campestris* L. ssp. *chinensis* var. *utilis* Tsen et Lee) is a subspecies of Chinese cabbage originally from Southern China that is now planted throughout the country, due to increasing consumer demand^3^. The major food product of flowering Chinese cabbage is the stalk, the development of which is directly related to plant quality and yield. Stem elongation, thickening, and flowering are key characteristics of flowering Chinese cabbage stem development, but the molecular mechanisms underlying this development are not well understood. Previously, cloned dihydroflavonol-4-reductase-like/UDP-d-apiose/UDP-d-xylose synthase genes by cDNA-amplified fragment length polymorphism analysis of bolting or flowering in flowering Chinese cabbage, but the role of these genes in floral organ development remain unclear^25^. Bolting-associated genes in flowering Chinese cabbage—including *FCA*, *FVE*, *FLOWERING LOCUS D* (*FLD*), and *FLOWERING LOCUS C* (*FLC*)—have also been cloned; the main pathways controlling flowering in *B*. *campestris* may be autonomous, distinct from vernalization-associated pathways, and dependent on *FRIGIDA* (*FRI*)^26^.

Stem development is a complex process that cannot be completely elucidated by traditional molecular approaches, such as gene cloning. Transcriptome analysis enables the global characterization of phenotypes in organisms^27^ and has been widely used to study stem and organ development, and the regulation of flowering in species such as tuberous mustard^7^, lotus (*Nelumbo nucifera*)^28^, radish (*Raphanus sativus*)^29^, and *B*. *napus* ^30^. Characterizing stalk development at the molecular level can provide information that is applicable for crop improvement.

To this end, the present study identified genes that were differentially expressed during three stages of stalk development (seedling, bolting, and flowering) in flowering Chinese cabbage by RNA sequencing (RNA-seq). These results provide insight into the molecular mechanisms of stalk development of flowering Chinese cabbage and other stalk vegetables.

## Results

### Flowering Chinese cabbage stalk development and sampling

**Figure 1:**
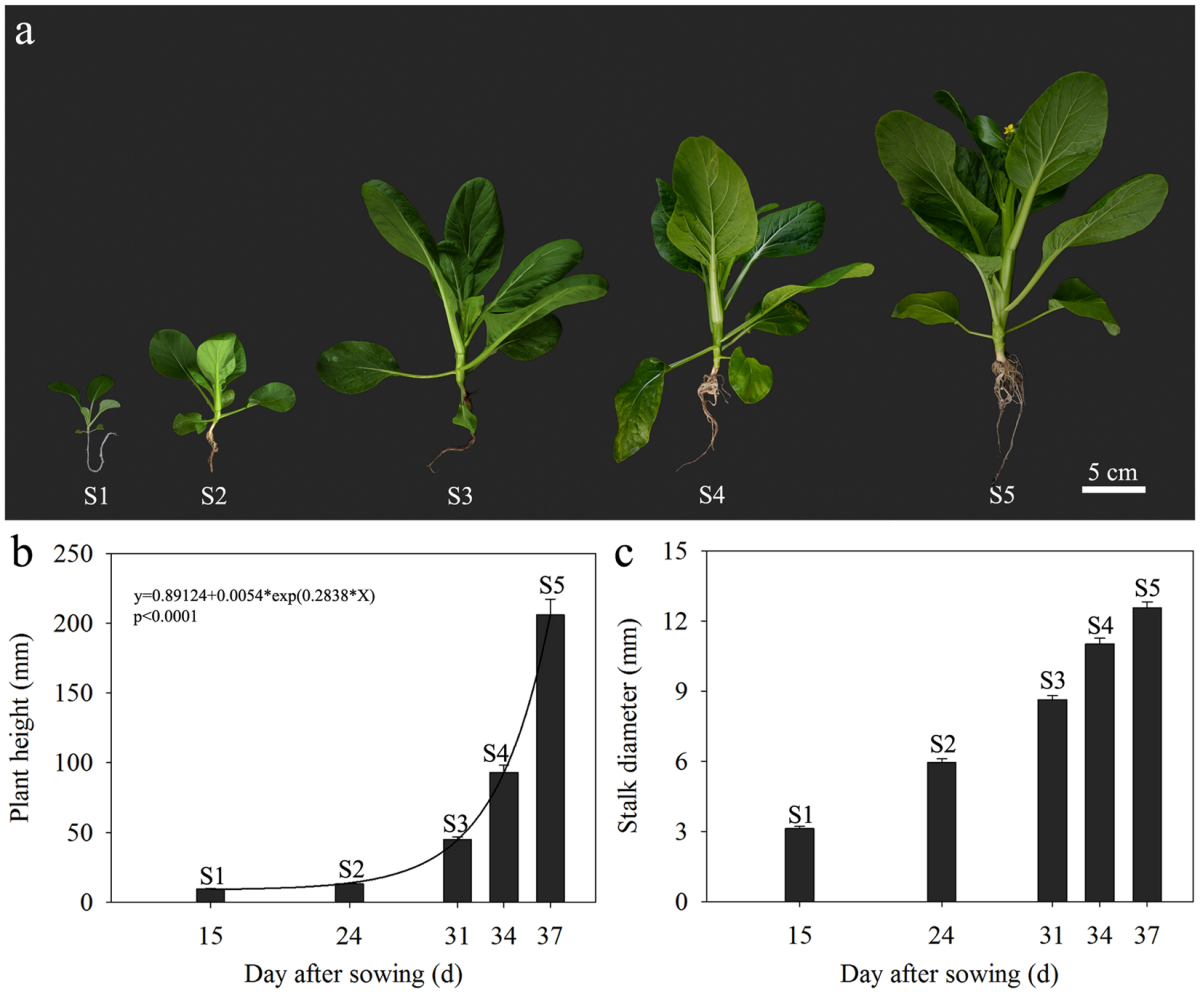
Stages of stalk development in flowering Chinese cabbage. (**a**) Plant morphology at different stages (S1 to S5). (**b**) Plant height at different stages; curve fitting was performed for the plant height at each sampling time (i.e., days after sowing), which is presented on the X-axis. (**c**) Stalk diameter at different stages, according to the sampling time (i.e., days after sowing), which is presented in the X-axis. Error bars indicate standard errors.

Bolting is the most obvious morphological characteristic of flowering Chinese cabbage. Based on bolting, stalk development can be classified into five stages (Fig. 1a). S1 (15 d after sowing, the seedling stage) encompasses the time from germination to the growth of three true leaves. S2 (24 d after sowing, the leaf-growing stage) is characterized by an increase in the leaf number and area. S3 (31 d after sowing, the bolting stage) represents the turning point of bolting, as the stalk bolts fast and continues to thicken. S4 (34 d after sowing, the budding stage) is the stage at which flower buds appear, while stalks continue to elongate and thicken. S5 (37 d after sowing, the flowering or harvesting stage) is characterized by stalks with one or two florets; plant quality is maximal at this stage. Figure 1b shows that plant height rapidly increased during S3/S4 and that the stalks showed an exponential elongation pattern during these development stages; curve fitting showed that S3 was the turning point of bolting. Stalk diameters showed a steady growth trend during S1–S5 (Fig. 1c). In this study, we focused on transcriptional changes in shoot tips at three representative periods, namely S1, S3, and S5 (Supplementary Fig. S1).

### Analysis of stalk anatomy

**Figure 2:**
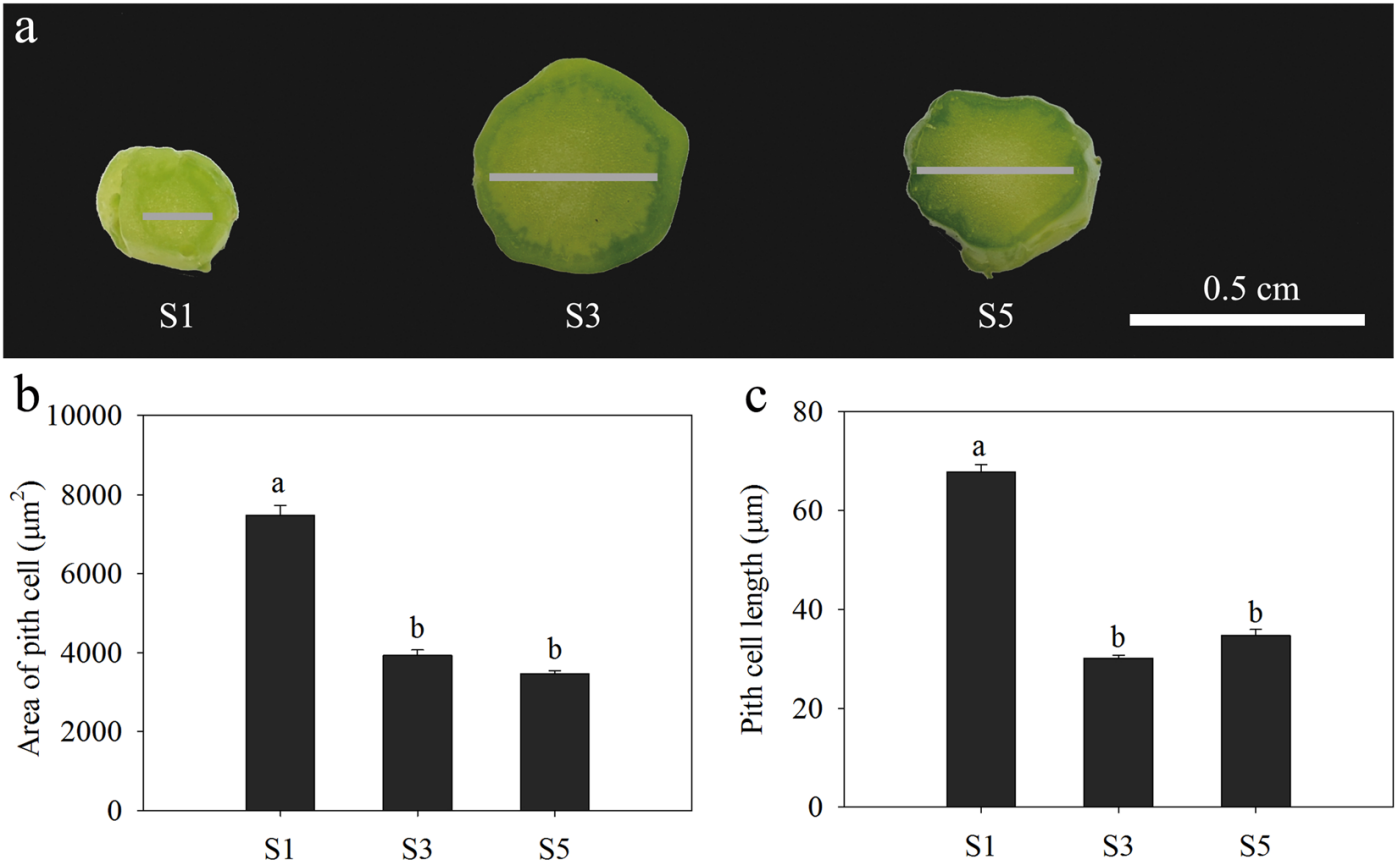
Analysis of stalk anatomy at three stalk development stages (S1, S3, and S5) of flowering Chinese cabbage. (**a**) Transverse sections of shoot tips at the different developmental stages; grey lines denote the pith region. (**b**) Area of pith cells in transverse sections at each developmental stage. (**c**) Pith cell lengths in longitudinal sections at each developmental stage. Values with different letters indicate significant differences at P < 0.05 according to Duncan’s multiple-range tests. Error bars indicate standard errors.

We measured changes in stalk diameter from transverse sections of samples obtained at different developmental stages of flowering Chinese cabbage. The pith region occupied most of the stalk, whose enlargement mostly resulted from a rapid increase in pith tissue growth (Fig. 2a). The area of pith cells declined over the course of development (Fig. 2b), whereas an analysis of longitudinal sections revealed that pith cell length decreased during bolting (Fig. 2c).

### Overview of RNA-seq

**Figure 3:**
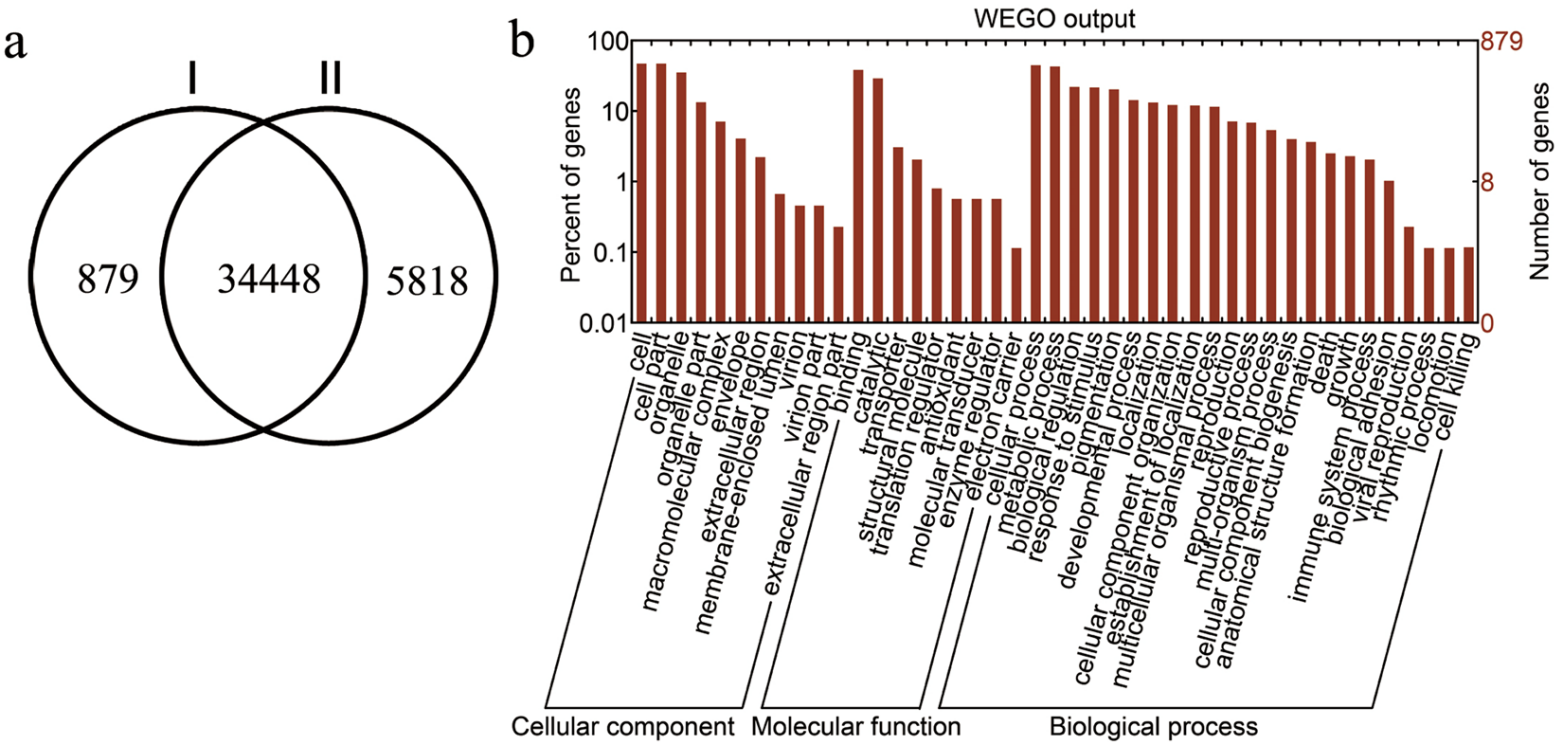
Novel transcripts identified in the flowering Chinese cabbage transcriptome. (**a**) Comparison between genes from our dataset (I) and the *Brassica rapa* genome database (II). (**b**) Gene ontology classification of novel transcripts.

RNA-seq was performed using two biological replicates of shoot tip specimens from S1, S3, and S5, generating six libraries (Table 1). Among the 16.3 G raw reads generated, 138 million high-quality (Q > 20) 125 bp reads were selected for further analysis (Table 1). After removing rRNAs by comparison with the rRNA database, 74.82%–76.22% of the short clean reads were uniquely mapped to the *B*. *rapa* genome (v.1.0). A total of 34,448 known genes identified by mapping to the *B*. *rapa* genome (Fig. 3a) were found in each of the six libraries: 28,730, 28,562, 29,393, 27,913, 31,386, and 32,158 (Table 1). Annotation using the NCBI Non-Redundant Protein Database (Supplementary Table S1) enabled identification of 879 novel transcripts (among which, 573 were common in all six libraries, and the remaining transcripts were isolated from one or more libraries). Of these, 415 were categorized into three main gene ontology (GO) categories, including 23 biological processes, nine molecular functions, and 11 cellular components (Fig. 3b). In the biological processes category, the most frequent distributions were ‘cellular processes’ (44.6%), ‘metabolic processes’ (42.7%), and ‘biological regulation’ (22.0%). Within the molecular functions category, transcripts were mostly distributed in ‘cell’ (47.0%), ‘cell part’ (47.0%), and ‘organelle’ (35.9%). Most transcripts within cellular components were classified into ‘binding’ (38.1%) and ‘catalytic activity’ (28.9%). We further compared the expression levels of the novel and annotated transcripts, using fragments per kilobase of transcript per million fragments (FPKM) values. Cumulative distributions revealed that the FPKM values of novel transcripts were mainly distributed below 5 (>73.72%), and half of the all transcripts (including novel and known transcripts) were >5 (Supplementary Fig. S2a–c). In addition, we compared the differential expression of novel transcripts during the S1, S3, and S5 periods, finding that the number of up-regulated genes in the S5 period was higher than those in other two periods (Supplementary Fig. S2d).

**Table 1:** Summary statistics for flowering Chinese cabbage clean reads in the six libraries mapped to the *Brassica rapa* reference genome.

Stage	S1		S3		S5	
Library	S1-1	S1-2	S3-1	S3-2	S5-1	S5-2
Clean reads	21,635,728	21,942,576	25,842,072	20,554,776	23,718,748	24,395,642
High quality clean reads (%)	21,630,166 (99.97%)	21,934,758 (99.96%)	25,833,896 (99.97%)	20,548,120 (99.97%)	23,707,618 (99.95%)	24,387,698 (99.97%)
Removed rRNA reads (%)	18,923,678 (87.49%)	19,461,054 (88.72%)	22,791,392 (88.22%)	18,349,778 (89.30%)	18,927,670 (79.84%)	21,879,804 (89.72%)
Mapped reads (%)	143,837,93 (76.01%)	14,794,144 (76.02%)	17,225,475 (75.57%)	13,855,253 (75.51%)	14,476,371 (76.48%)	16,841,272 (76.97)
Unique mapped reads (%)	14,269,607 (75.41%)	14,676,810 (75.42%)	17,054,871 (74.83%)	13,728,653 (74.82%)	14,329,425 (75.71%)	16,676,832 (76.22%)
Multiple mapped reads	114,186 (0.60%)	117,334 (0.60%)	170,604 (0.75%)	126,600 (0.69%)	146,946 (0.78%)	164,440 (0.75%)
All genes	29,445	29,274	30,127	28,612	32,169	33,006
Known genes (%)	28,730 (71.35%)	28,562 (70.93%)	29,393 (73.00%)	27,913 (69.32%)	31,386 (77.95%)	32,158 (79.86%)
Novel transcripts	715	712	734	699	783	848

### Differentially expressed genes in the three stages of stalk development

We analysed correlations between the six samples using FPKM and Pearson’s correlation coefficients (R^2^); the latter were greater than 0.90 (using R^2^ > 0.8 as the significance cut-off) between the two replicates of each stage, indicating we could use both samples from each stage (Supplementary Fig. S3). The R^2^ values among S1, S3, and S5 groups were lower than 0.77, indicating that gene expression levels differed among the three stages (Supplementary Fig. S3).

**Figure 4:**
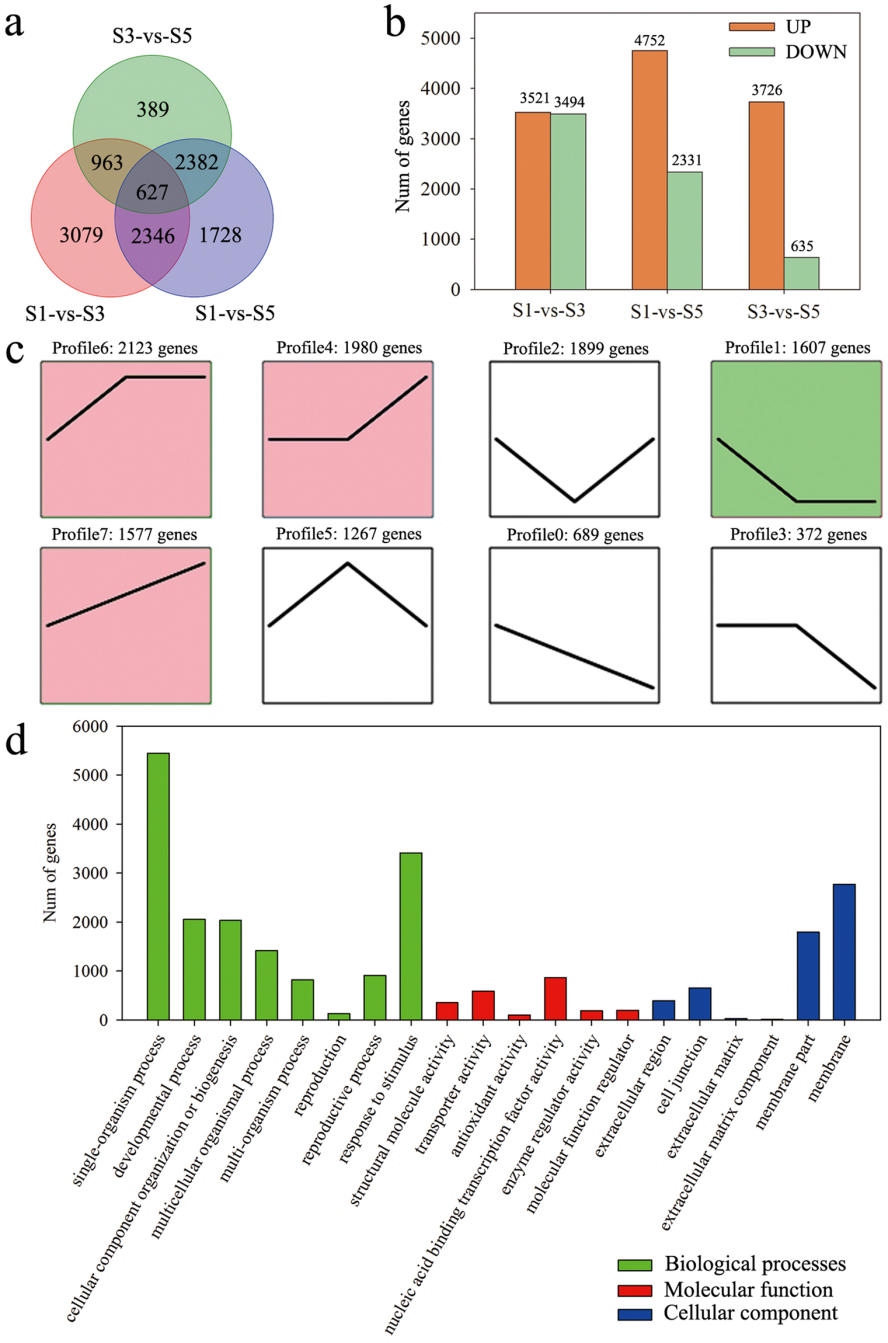
Analyses conducted for the DEGs identified by pairwise comparisons among three stages of stalk development (S1, S3, and S5) in flowering Chinese cabbage. (**a**) Venn diagram of the number of DEGs in the three stages. (**b**) Pairwise comparisons of gene expression. The number of up- and down-regulated genes in each pair of stages is presented. (**c**) Gene-expression patterns in the eight major profiles; the colours in each profile indicates their statistical significance (P ≤ 0.05) (red, up-regulated; green, down-regulated). (**d**) Gene Ontology terms (q ≤ 0.05) significantly enriched by DEGs.

To identify genes associated with stalk development, the expression of each gene within the library from each developmental stage was compared to that of other developmental stages (pairwise comparisons) and then filtered using |log2 (fold-change)| > 1 and a false-discovery rate (FDR) < 0.05. These pairwise comparisons allowed identification of 11,514 differentially expressed genes (DEGs) at the three stages—i.e., 7,015, 7,083, and 4,361 DEGs from S1 vs. S3, S1 vs. S5, and S3 vs. S5, respectively (Fig. 4a). In addition, 627 genes showed significantly different expression among the three stalk developmental stages. A total of 2,346 genes in S1, 963 genes in S3, and 2,382 genes in S5 showed significantly different expression, compared with the two other developmental stages. In S1 vs. S3, 3,521 and 3,494 genes were up- and down-regulated, respectively; in S1 vs. S5, 4,752 and 2,331 genes were up- and down-regulated, respectively; and in S3 vs. S5, 3,726 and 635 genes were up- and down-regulated, respectively (Fig. 4b).

The 11,514 DEGs were clustered into eight profiles by STEM (Short Time-series Expression Miner), including four significant expression profiles (profiles 1, 4, 6, and 7) (Fig. 4c); the heatmap of all DEGs showed the same clustering pattern (Supplementary Fig. S4). Profile 6 included 2,123 genes that were rapidly up-regulated at S3 but showed a constant level of expression at S5; 1,980 genes from profile 4 showed no difference between S1 and S3, but were up-regulated at S5; in profile 1, 1,607 genes were down-regulated at S3 that showed no difference at S5; and DEGs in profile 7 were up-regulated at both S3 and S5.

### GO and pathway-enrichment analyses of DEGs in stalk development

We classified the functions of all DEGs in the three developmental stages by GO assignment. Twenty subcategories were significantly enriched for three GO functional categories. Among these subcategories, the GO terms ‘single-organism process’, ‘nucleic acid binding transcription factor activity’, and ‘membrane’ were the most significantly enriched in biological processes, molecular functions, and cellular components, respectively (Fig. 4d). Moreover, some significantly enriched GO terms in biological processes were related to plant flowering and morphogenesis, including ‘anatomical structure morphogenesis’, ‘pollen development’, ‘cell differentiation’, ‘floral organ development’, ‘flower development’, ‘cell division’, ‘cell cycle process’, and ‘floral organ morphogenesis’ (Supplementary Table S2).

We further analysed GO terms that were significantly enriched for in the biological processes category within the four significant expression profiles (Supplementary Table S3). In profile 6, the most significantly enriched GO terms were involved in the regulation of gene expression, including ‘RNA modification’, ‘gene expression’, ‘nucleobase-containing compound metabolic process’, and ‘nucleic acid metabolic process’. Processes related to cell wall organization or biogenesis, cell morphogenesis, development, and differentiation, such as ‘plant-type cell wall organization’, ‘plant-type cell wall organization or biogenesis’, ‘cell morphogenesis’, ‘cell development’, and ‘cell differentiation’, were significantly enriched in profile 4. In profile 1, down-regulated genes were significantly enriched in processes related to stress responses and nutrient levels, such as ‘response to reactive oxygen species’, ‘response to oxidative stress’, and ‘cellular response to nutrient levels’. Up-regulated genes in profile 7 were significantly enriched in processes associated with cellular development and morphogenesis, including ‘cellular component assembly involved in morphogenesis’, ‘extracellular matrix assembly’, and ‘cellular developmental process’.

To further understand their putative active biological pathways, all identified DEGs were mapped to the Kyoto Encyclopaedia of Genes and Genomes (KEGG) database, and pathways with q value ≤ 0.05 were considered significantly enriched. Overall, 2,625 DEGs were assigned to KEGG pathways and 15 KEGG pathways were significantly enriched (Supplementary Fig. S5). ‘Ribosome’ (n = 357, 13.6%), ‘plant hormone signal transduction’ (n = 211, 8.04%), ‘starch and sucrose metabolism’ (n = 147, 5.6%), ‘phenylpropanoid biosynthesis’ (n = 132, 5.03%), and ‘pentose and glucuronate interconversions’ (n = 78, 2.97%) were the main significantly enriched pathways. An analysis of the distribution of ‘plant hormone signal transduction’ genes in each profile revealed that this pathway was significantly enriched in profiles 0, 1, and 2 (Supplementary Fig. S6).

### Expression patterns of genes encoding plant hormone-signalling components and cell cycle genes regulated by plant hormones

**Figure 5:**
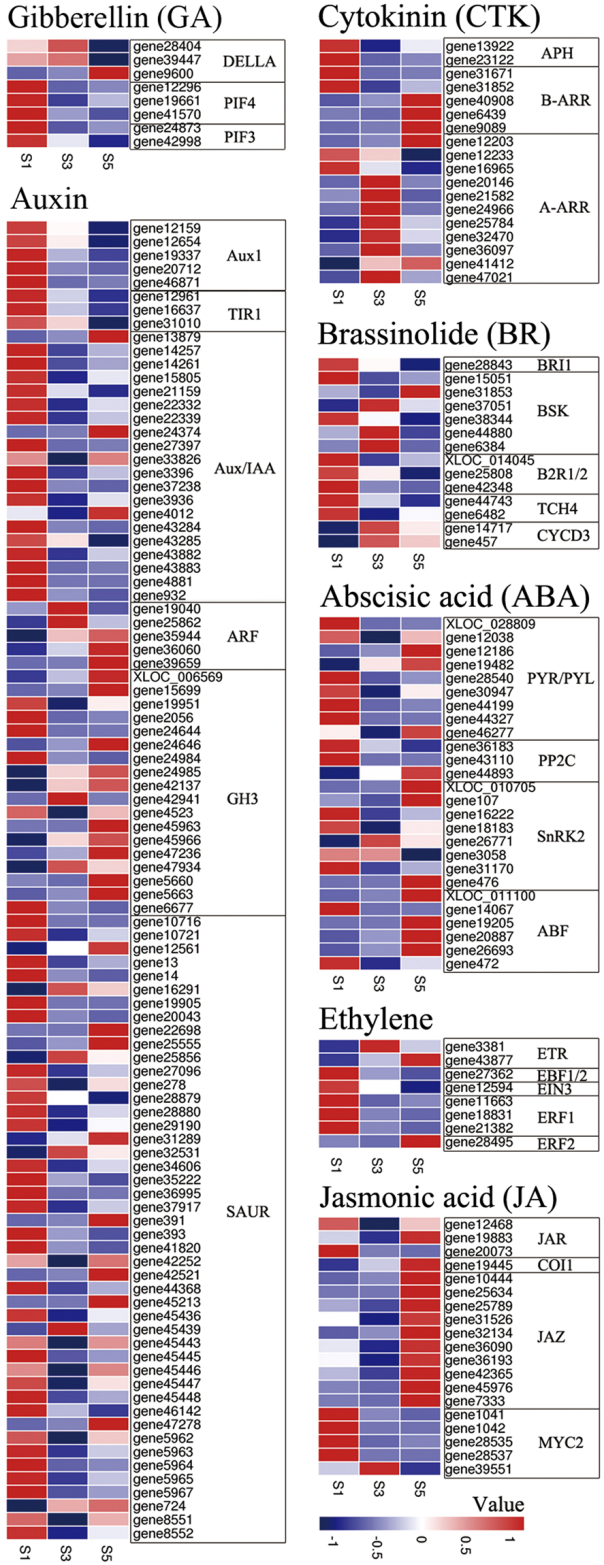
Heat map of DEGs assigned to plant hormone signal transduction pathways. Values represent the Z-scores of FPKM. Red and blue colours represent up- and down-regulation, respectively.

Many DEGs were related to hormone signal transduction (Fig. 5), including eight DEGs associated with GA signal transduction and two DELLA homologues (*REPRESSOR OF GA1* [*RGA1*] and *RGA2-like*) that were down-regulated at S5. Ninety-seven genes in the auxin signalling pathway were differentially expressed, including three *Aux*/*IAA*, five *AUXIN RESPONSE FACTOR* (*ARF*), twelve GRETCHEN HAGEN 3 (*GH3*), and twelve SMALL AUXIN UPREGULATED RNA (*SAUR*) genes that were up-regulated at S3 and/or S5. Two genes encoding aminoglycoside phosphotransferase (*APH*) and two genes encoding *Arabidopsis* type B and type A cytokinin response regulators (*B-ARR* and *A-ARR*, respectively), which are involved in CTK signalling, were down-regulated during stalk development; however, three *B-ARR* and nine *A-ARR* were up-regulated at S3 and/or S5. Fourteen genes in the BR signal transduction pathway were differentially expressed, including four *brassinosteroid signaling kinase* (*BSK*) and two *CYCD3* genes that were up-regulated at S3 and/or S5. In addition, 12 genes related to ABA signalling were up-regulated at S3 and/or S5 and 14 were down-regulated, whereas eight genes related to ethylene signalling—of which five were down-regulated—were differentially expressed during stalk development. In the jasmonate (JA) signalling pathway, one *JASMONATE RESISTANT* (*JAR*), one *CORONATINE INSENSITIVE1* (*COI1*) and 10 *JASMONATE ZIM-Domain* (*JAZ*) genes were up-regulated at S5, and four *myelocytomatosis viral oncogene 2* (*MYC2)* genes were down-regulated during stalk development, with one *MYC2* gene down-regulated at S5.

**Figure 6:**
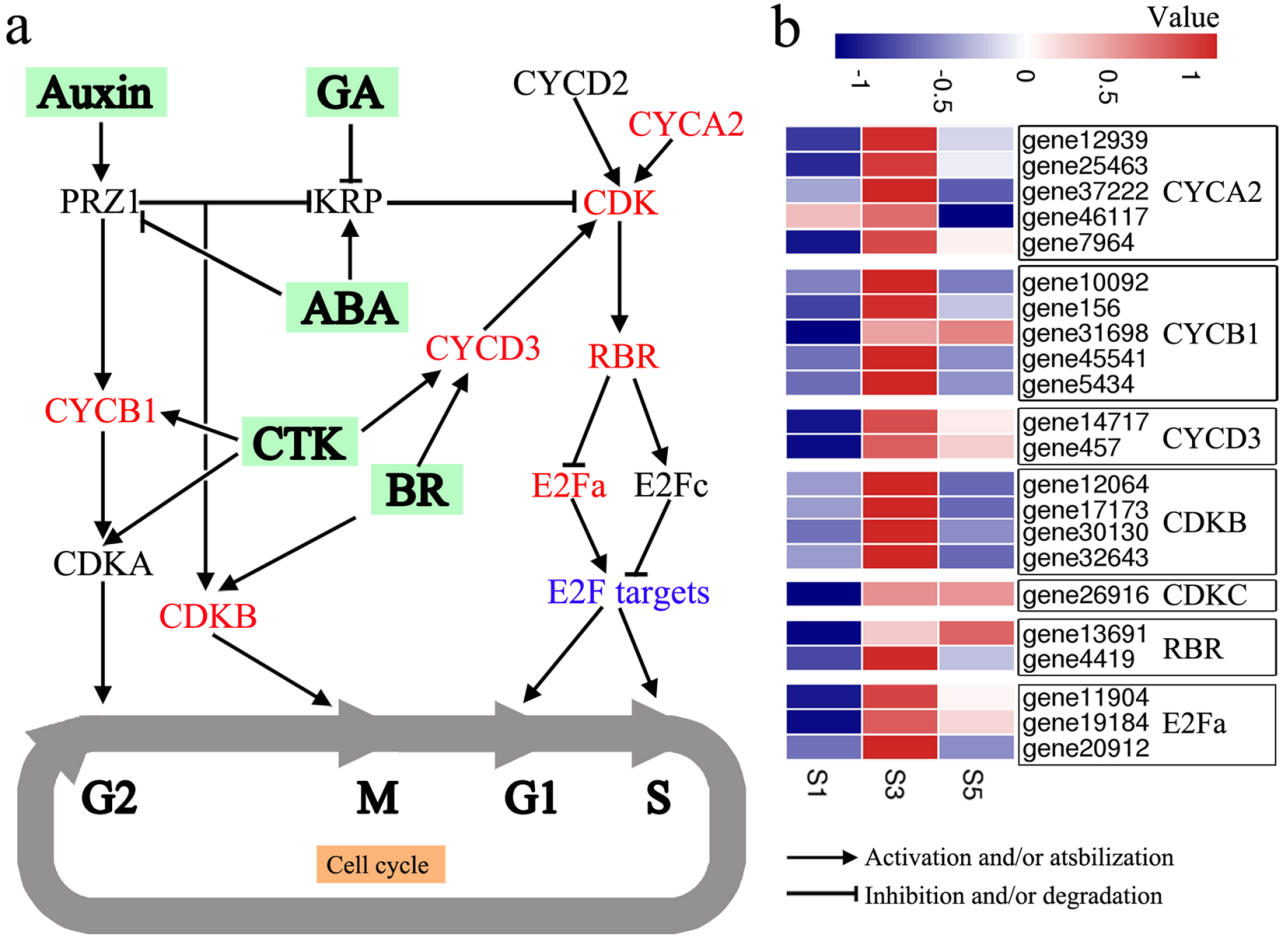
Signalling pathways related to cell cycle regulation. (**a**) Schematic representation of the cell cycle-regulation network. The expression of genes indicated in red were significantly different among the three stages of stalk development. Genes in black were not significantly different among the three stages of stalk development. Arrows represent the promotion of gene activation, and blunted lines represent gene repression. (**b**) Heat map of the DEGs associated with cell cycle regulation. Red and blue colours represent up- and down-regulation, respectively. The values represent the Z-scores for the FPKM data.

Plant patterning, growth, and development are dependent on the cell cycle, which is regulated by plant hormones. We analysed the anatomical characteristics of flowering Chinese cabbage shoot tips and found that cell division plays an important role in stalk development. We identified 22 DEGs related to cell cycle such as *CYCs*, *CDKs*, retinoblastoma-related (*RBR*) proteins, and *E2Fs* that were up-regulated at S3 (Fig. 6).

### Expression patterns of genes controlling flowering time

**Figure 7:**
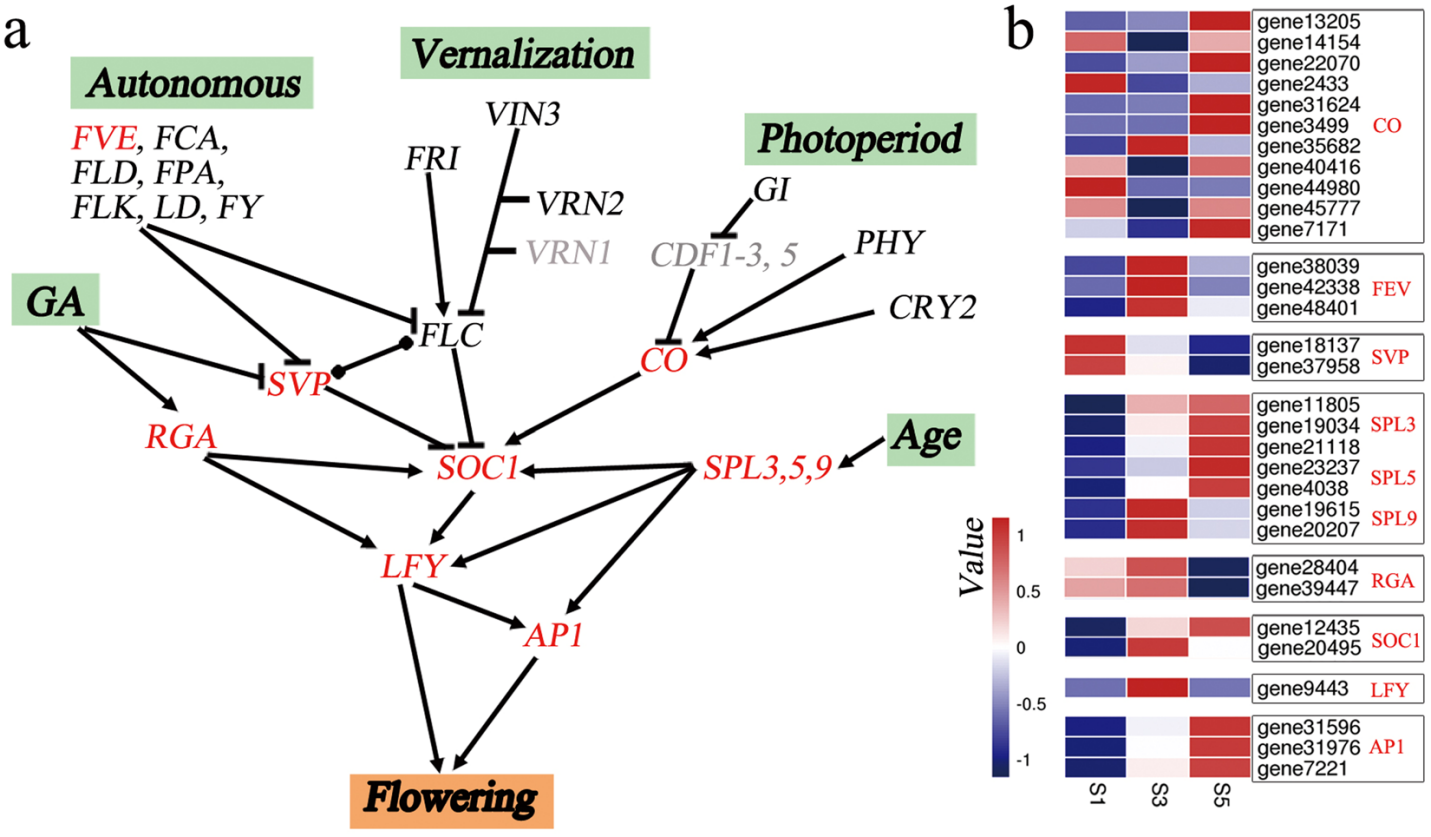
Signalling pathways related to the flowering time. (**a**) Simplified regulatory network of flowering time-related genes in the shoot apex. The expression levels of genes indicated in red were significantly different during the three stages of stalk development. The expression of genes in black were not significantly different during the three stages of stalk development. No significant differences in genes in grey were observed during the three stages of stalk development. Arrows represent the promotion of gene activation, blunted lines represent gene repression, and round dots at both ends mark an interaction without a known direction. (**b**) Heat map of genes that were differentially expressed during the three stalk development stages (S1, S3, and S5). Red and blue colours represent up- and down-regulation, respectively. The values represent the Z-scores of the FPKM data.

Many genes involved in the regulation of flowering time have been identified in *Arabidopsis* ^20^. These belong to six major flowering pathways and are expressed in the leaf and/or shoot apex. Based on the flowering-time regulation network in the *Arabidopsis* ^21^ and *Brassica* Database, we found that 147 genes were previously identified (Supplementary Table S4). We further screened 31 key flowering time-related genes that were differentially expressed in the shoot apex; most of these were associated with the GA, autonomous, photoperiod, and age pathways (Fig. 7). *RGA* genes (genes28404 and genes39447), which encode DELLA proteins and act as repressors in the GA pathway, were down-regulated at S5. *SHORT VEGETATIVE PHASE* (*SVP*) genes (genes18137 and genes37958) encode repressors and were down-regulated during stalk development. In the ‘autonomous’ pathway, three homologues of the gene encoding the activator FVE (genes38039, genes42338, and genes48401) were up-regulated at S3. Eleven homologues of CONSTANS (*CO*) were identified, most of which were up-regulated at S5. In the ‘age pathway’, the key activator genes *SQUAMOSA PROMOTER-BINDING PROTEIN-LIKE3* (*SPL3*), *SPL5*, and *SPL9* were up-regulated at S3 and S5. In addition, the flowering time-integrator genes *SUPPRESSOR OF OVEREXPRESSION OF CO 1* (*SOC1*), *LEAFY* (*LFY*), and *APETALA1* (*AP1*) were up-regulated at S3 and/or S5.

### Validation of RNA-seq results by quantitative real-time (qRT)-PCR

**Figure 8:**
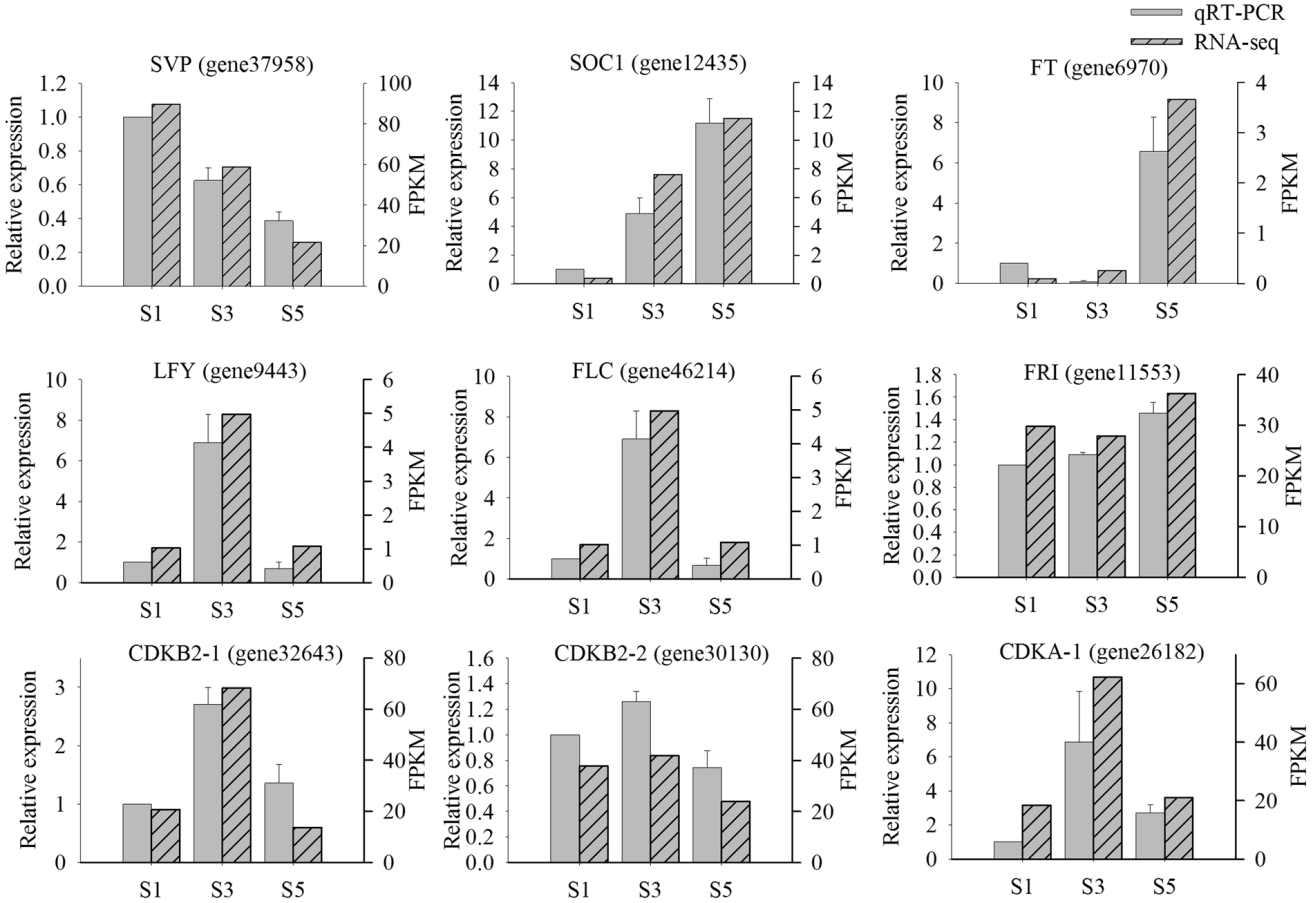
qRT-PCR validation of nine genes differentially expressed during the three stalk development stages (S1, S3, and S5), identified by RNA-seq. The left and right Y-axes indicate relative gene-expression levels determined by qRT-PCR and the FPKM obtained by RNA-seq, respectively. The results for each gene are based on three biological and three technical replicates. The error bars indicate standard errors.

To confirm the accuracy and reproducibility of RNA-seq expression profiles, we amplified nine genes by qRT-PCR using specific primers (Supplementary Table S5). Of these nine genes, six and three are known to control flowering time and cell cycle, respectively (Fig. 8). Their relative expression levels were analysed with the 2^−ΔΔCT^ method^31^, using glyceraldehyde 3-phosphate dehydrogenase (*GADPH*) as the reference gene^32^. All nine genes had the same expression pattern (Fig. 8), and the Pearson’s correlation coefficient between the RNA-seq and qRT-PCR data was 0.9127 (P < 0.0001), indicating the reliability of the RNA-seq data (Supplementary Fig. S7).

## Discussion

Flowering Chinese cabbage is a subspecies of Chinese cabbage with a bolting flower stalk as the major food product^26^. Stalk development in flowering Chinese cabbage is a complex process that includes thickening, elongation, and flowering. To clarify the molecular mechanisms of stalk development in this study, we used RNA-seq to compare gene-expression profiles at three different stages of this process.

Stem thickening and elongation is controlled via cell cycle regulation in meristematic regions^33,34^. We analysed cell alignment in transverse sections and found that stalk thickening mainly resulted from an enlargement of pith tissue (Fig. 2a) and that pith cell area decreased with increasing stalk diameter (Fig. 2b). These findings indicate that flowering Chinese cabbage stalk thickening mainly occurs via cell division in the intercalary meristem, similar to stem swelling in tuber mustard^34^. Stem elongation depends on increased cell division in the meristem and cell elongation in elongation zone^35,36^. In this study, pith cell length in longitudinal sections declined during flowering Chinese cabbage bolting (Fig. 2c), suggesting that cell division in shoot tip meristem is the major factor promoting stalk elongation in this plant.

Plant growth and development in *Brassica* are complex processes that can be understood by analysing gene expression and associated regulatory mechanisms^6,7,30^. In this study, we obtained 16.3 G raw reads from three stages of stalk development by RNA-seq, of which 75.40% were mapped to the reference genome of *B*. *rapa* (Table 1); this was higher than that obtained for the Chinese cabbage leaf transcriptome^37^, and the match scores fit the technological criterion. Up to 97.5% of the genes in this study were identified by mapping to the *B*. *rapa* genome; this high proportion indicates that both the sequences and the reference genome were suitable for further analysis. There were 35,327 genes that were previously predicted in the *B*. *rapa* genome (Fig. 3a) and 879 novel transcripts (Supplementary Table S1); the latter were identified by BLASTx and annotated based on four public databases. Analysis of the functional categories of novel transcripts revealed that ‘cellular processes’, ‘metabolic processes’, and ‘single-organism processes’ were the most common categories of biological processes. This GO information will help to further study novel transcripts. Analysis of the FPKM cumulative distributions showed that the expression of novel transcripts was lower, and that their FPKM values were mainly distributed below 5 (Supplementary Fig. S2), which is consistent with previous RNA-Seq data for Chinese cabbage^38^. The low expression of these novel transcripts might explain why they were not detected and annotated in previous studies. In addition, the expression of these novel transcripts in S5 exceeded that in S1 and S3 (Supplementary Fig. S2d), suggesting that these novel transcripts might be related to stalk development.

To identify genes that regulate stalk bolting, we compared the transcriptional profiles of stalks at three developmental stages. We found 7,015, 7,083, and 4,361 DEGs in pairwise comparisons of S1 vs. S3, S1 vs. S5, and S3 vs. S5, respectively (Fig. 4a). To further understand gene functions and putatively active biological pathways, all DEGs identified were mapped to the KEGG and GO databases. Many DEGs corresponding to starch and sucrose metabolism, and plant hormone signal transduction were detected (Supplementary Fig. S5). In addition, many GO terms associated with flowering and morphogenesis were significantly enriched, including terms related to floral organ development and morphogenesis, pollen development, and cell division and differentiation (Supplementary Table S2).

We clustered DEGs into eight profiles by STEM and found that four were significantly expressed (Fig. 4c). Profile 6 was markedly up-regulated at S3; significantly enriched GO terms at this stage were associated with the regulation of gene expression, including ‘RNA modification’, ‘gene expression’, ‘nucleobase-containing compound metabolic process’, and ‘nucleic acid metabolic process’. Because S3 is the turning point period of flowering Chinese cabbage bolting, the initiation of bolting seems to be related to these processes. Marked changes in morphology occur from S3 to S5 in flowering Chinese cabbage, including flower stalk development and organ formation; gene expression in profiles 4 and 7 correlated with these changes. Profile 4 genes were mainly related to cell wall and cell morphogenesis, development, and differentiation. The cell wall contributes to plant form and function, and affects plant growth; for example, cell wall relaxation is a prerequisite for cell growth^39^. In profile 7, up-regulated genes were enriched in processes concerning cellular development and morphogenesis such as ‘cellular component assembly involved in morphogenesis’, ‘extracellular matrix assembly’, and ‘cellular developmental process’. Therefore, the expression profiles were in agreement with the morphological findings.

Environmental factors and endogenous signals coordinately regulate plant growth and development. Phytohormones are endogenously occurring compounds that transmit environmental signals to intracellular signalling pathways and regulate multiple aspects of plant growth and development, including stalk development^40,41^. In our study, several hormones signalling pathway were significantly enriched by pathway-based analysis (Fig. 5). DELLA domain proteins are transcriptional regulators that respond to GA and are GA-induced repressors of growth and flowering^42–44^. Two DELLA homologues, *RGA1* (gene28404), and *RGA2-like* (gene39447), were down-regulated during stalk development (Fig. 5), and the lack of RGA function may advance flowering and promote stem growth^43,44^, suggesting that stalk development in flowering Chinese cabbage is likely regulated by *RGA* via GA signalling. The auxin-response factors Aux/IAA and ARF transcription factors can mediate auxin responses and modulate stalk development by regulating cell division^45,46^, and ARFs bind to TGTCTC auxin-response elements in the target gene promoter to activate or inhibit their transcription, which can be repressed by Aux/IAA^47^. In our study, five *ARF* genes were up-regulated and 18 *Aux/IAA* genes were down-regulated at stages S3 or S5 of stalk development (Fig. 5), suggesting that auxin-related genes play critical roles in stalk development. *A-ARR* and *B-ARR* genes play different roles in CTK signalling: *A-ARRs* negatively regulate cytokinin responses and *B-ARRs* promote cytokinin-regulated gene expression^48^. Eight *A-ARR* and three *B-ARR* genes were more highly expressed at S3 and S5, respectively (Fig. 5). Nevertheless, the role of these two kinds of CTK signal-transduction genes in stalk development needs to be verified. *CYCD3* is a D-type CYC that is regulated by BR to stimulate cell division^49^, and two *CYCD3* genes (genes 457 and 14,717) were up-regulated at the bolting stage (i.e., S3; Fig. 5). ABA signalling was previously linked to stem inflation in tuberous mustard^15^ and in this study, four homologues of *ABF*—XLOC011100 and genes19205, genes20887, and genes26693—were up-regulated, indicating that ABA signalling likely regulates stalk thickening in flowering Chinese cabbage. We also identified other plant hormone-responsive genes such as *ethylene receptor* (*ETR*), eukaryotic release factor 1/2 (*ERF1/2*), *JAZ*, and *MYC*. Overall, these results indicate that plant hormones are crucial for stalk development in flowering Chinese cabbage and that they cooperate to regulate stalk development.

Anatomical findings show that cell division is the major factor promoting stalk thickening and elongation in flowering Chinese cabbage. Cell division in plants is regulated by hormones through a core group of cell cycle-related genes^36,37^, including *CYC*, *CDK*, *RBR*, and *E2F*. *CDK* activity is a core feature of cell cycle control in eukaryotes^17^; for instance, *CDKB1* is expressed during the G2-to-M transition under the control of auxin^50^. CYCs combine with and modulate the activity of CDKs to regulate cell cycling^18^. E2F promotes DNA replication^18^, which is inhibited by RBR to maintain a balance between cell proliferation and differentiation^51^. CYC–CDK complexes phosphorylate RBR, which releases E2F from this inhibition. In our study, *CYCD3*, *CYCA2*, *CYCB1*, *CDKB*, *CDKC*, *RBR*, and *E2Fa* were up-regulated during stalk development, mainly at the bolting stage (Fig. 6), suggesting that these cell cycle-related genes regulate cell division during stalk development.

Bolting and flowering are interdependent processes. The floral organ develops during the bolting period, and the flowering transition time affects the bolting time. Over 200 genes that regulate the flowering time have been isolated in *A*. *thaliana* ^19^. In our study, 147 genes related to the flowering time were identified in six pathways (Supplementary Table S4), with 31 genes differentially expressed in the shoot apex, including *RGA*, *SVP*, and *FVE* (Fig. 7). In our transcriptome data, vernalization-related genes showed stable expression patterns (Fig. 7), and this finding was consistent with flowering Chinese cabbage growth. *FVE* is a component of the autonomous pathway^52^ and was up-regulated during stalk development (Fig. 7), suggesting that the flowering time in flowering Chinese cabbage is controlled by the autonomous pathway, as previously demonstrated^26^. However, the observed level of *FLC* expression differed from that reported earlier^26^, which might be explained by the epigenetic regulation of *FLC* by *FVE* via histone deacetylation, as opposed to transcriptional regulation^53^. Gibberellins promote the expression of both *SOC1* and *LFY* by inducing the degradation of growth-suppressing DELLA proteins in the shoot apex^54,55^. Genes28404 and genes39447 are *RGA* homologues that encode DELLA proteins and were down-regulated during stalk development (Fig. 7). SVP mediates flowering in response to ambient temperature via GA signaling^56,57^ and regulates the expression of flowering-integrator genes such as *SOC1* in leaves or shoot apex genes that promote flowering^58^. We found that *SVP* was down-regulated during stalk development in the former but was unaltered during this period in the latter (Fig. 7). Thus, these results suggest that GA signalling modulates the flowering time in *B*. *campestris*.

Flowering Chinese cabbage is insensitive to the photoperiod and can be planted throughout the year in Southern China. This characteristic might explain why photoperiod-responsive genes were not differentially expressed during stalk development. Nonetheless, 11 *CO* homologues showed differential expression in our study, which should be studied further. The age pathway ensures plant flowering under non-inductive conditions and is known to involve the microRNA miR156-SPL^59^. As plants age, miR-156 induces/inhibits *SPL* expression in the shoot apex. Up-regulated *SPL* levels activate flowering-time genes including *AP1*, *LFY*, and *SOC1* ^59,60^. Seven *SPL* genes were up-regulated in S3 and/or S5, including *SPL3*, *SPL5*, and *SPL9* (Fig. 7). *SPL3* or *SPL9* overexpression in the shoot apex can lead to an early-flowering phenotype under both long- and short-day conditions^60^. Thus, the ‘age pathway’ is critical for controlling the flowering time in *B*. *campestris*.

*SOC1*, *LFY*, and *AP1* are three major floral meristem regulators in *A*. *thaliana*. *SOC1* is an integrin that controls almost all flowering pathways to promote flowering; *LFY* indirectly and directly mediates flowering by interacting with *SOC1* and activating GA- and age-related signalling pathways; and *AP1* is targeted by *LFY* and *SPL* and by the FT protein expressed in leaves^20,61^. Overexpression of *B*. *rapa SOC1* in *B*. *napus* induced the expression of *LFY* and *AP1*, with transgenic plants exhibiting early flowering^62^. In our study, two *SOC1*, one *LFY*, and three *AP1* genes were up-regulated during stalk development (Fig. 7), suggesting they are key regulators of floral meristem development in flowering Chinese cabbage.

## Conclusions

Global transcriptional changes during stalk development in flowering Chinese cabbage were investigated for the first time by RNA-seq. In total, 11,514 genes were differentially expressed among the three stages of stalk development, with significant enrichment in the ‘ribosome’ and ‘plant hormone signal transduction’ pathways. The DEGs involved in hormone signalling, cell cycle progression, and regulation of the flowering time formed a regulatory network controlling stalk development. These results provide a basis for future studies on the molecular mechanisms of stalk development in flowering Chinese cabbage and other stalk vegetables, such as Chinese kale and purple cai-tai, and provides a new theoretical basis for breeding stalk vegetables.

## Methods

### Plant materials

The early-maturing variety of flowering Chinese cabbage ‘Youlv 501 caixin’ was used in this study. This variety was developed at the Guangzhou Institute of Agricultural Sciences (Guangzhou, China) and can be grown in plain regions of China from late September to early November. Experiments were performed in a greenhouse (25–30 °C, with natural sunshine) and in a field at the Guangdong Provincial Engineering Technology Research Center for Protected Horticulture (South China Agricultural University in Guangzhou, China). Sterile seeds were germinated in a climatic cabinet within the greenhouse and sown in plug trays, using perlite as the substrate. When plants grew three true leaves, seedlings were transferred to a potting mix consisting of peat, perlite, and coco peat (1 :1: 1). Plants were watered every few days with a half-dose of Hoagland formula. Plant heights and stalk diameters were measured with 30 plants; the former was the distance from the cotyledon to the shoot tip, and the latter was the diameter of the stalk segments between the fourth to fifth true leaves, except at S1 when the stalk diameter was determined by measuring the residual portion of the stalk. SigmaPlot v.11 software (SYSTAT, San Jose, CA, USA) was used to plot these data.

### Paraffin sections and imaging

Plant samples (shoot tip = 5 mm) of flowering Chinese cabbage at stages S1, S3, and S5 (Supplementary Fig. S1) were fixed in formalin–acetic acid–alcohol fixative solution (70% ethanol: formalin: glacial acetic acid, 18: 1: 1) at room temperature (22–25 °C) for 24 h, followed by dehydration in ethanol and embedding in paraffin. Sections were cut at a thickness of 8 µm on an RM2255 microtome (Leica, Wetzlar, Germany), stained with Safranin O and Fast Green, and imaged under bright-field illumination in an DM4000 B microscope (Leica). Image-Pro Plus v.6 software (Media Cybernetics, Rockville, MD, USA) was used to measure cell lengths in longitudinal sections and cell area in transverse sections. Pith cell lengths in longitudinal sections were measured in three to five random areas on each section (n > 50 for each section); pith cell areas in transverse sections were measured in five random areas on each section (n > 50 for each section)^63^. Means were compared by the Duncan’s multiple-range tests at P < 0.05. SigmaPlot v.11 software was used to create Fig. 2b,c.

### RNA extraction and sequencing

For transcriptome assembly, the shoot tips (5 mm) of 20 plants were pooled into a single sample at stages S1, S3, and S5 (Supplementary Fig. S1) and immediately flash frozen in liquid nitrogen and stored at −80 °C until use. Two biological replicates were prepared for each stage. The RNeasy Plant Mini Kit (Qiagen, Valencia, CA, USA) was used for total RNA extraction, according to the manufacturer’s protocol. DNase I (Takara Bio, Ostu, Japan) was used to digest genomic DNA, and RNA integrity was analysed on a 1% agarose gel and in a 2100 Bioanalyzer (Agilent Technologies, Santa Clara, CA, USA). RNA samples with high purity were used to prepare cDNA libraries^64^, and six cDNA libraries were sequenced using the HiSeq. 2500 system (Illumina, San Diego, CA, USA) at the Beijing Genomics Institute (Shenzhen, China), generating 125-bp paired-end reads.

### RNA-seq data analysis: mapping and differential expression

Before library assembly, raw reads were processed using in-house Perl scripts in fastq format, and were filtered using the FASTX Toolkit (http://hannonlab.cshl.edu/fastx_toolkit/) to remove adapter sequences and low-quality reads containing >50% of bases with Q ≤ 20 and/or >10% unknown (N) bases. The copyright for Perl script belongs to Guangzhou Genedenovo Biotechnology Co. Ltd (http://www.genedenovo.com/). High-quality clean reads were compared to those in the Ribosomal Database Project, and rRNAs were removed using Bowtie v.2.2.8^65^. Non-rRNA high-quality clean reads from all six libraries were merged and aligned to the *B*. *rapa* reference genome v.1.0 (http://brassicadb.org)^66^ using TopHat2 v.2.1.1^67^. Novel transcripts were identified from the TopHat2 alignment using the Cufflinks v.2.2.1 reference annotation-based transcript assembly method^28^. BLASTx was used to annotate novel transcripts based on the NCBI Non-Redundant Protein Database (www.ncbi.nlm.nih.gov).

FPKMs for each gene were calculated based on the gene length, and read counts were mapped to these genes^68^. Pearson’s correlation coefficients among the six samples were determined using R (http://www.r-project.org/). The DEGs between two samples were identified using the edgeR package v.3.12.1 (http://www.r-project.org/)^69^ by employing FDR < 0.05 and |log2 (fold-change)| > 1 as criteria for determining significant differences in gene-expression levels. A log2 (fold-change) > 1 indicates up-regulation and a log2 (fold-change) < −1 indicates down-regulation. STEM software (http://www.cs.cmu.edu/~jernst/stem/) was used to cluster DEGs according to temporal expression profiles. The maximum unit change in model profiles between time points was 1 and the minimum ratio of fold-changes in DEGs was no less than 2.0; profiles with P ≤ 0.05 were considered significantly enriched.

### GO enrichment and KEGG pathway analysis

GO and KEGG analyses were performed to identify significant enrichments in the GO terms and metabolic pathways of DEGs. The GOseq R package (http://www.r-project.org/) was used to analyse DEGs in terms of GO enrichment relative to the *B*. *rapa* genome background^70^, and KEGG pathway analysis was performed using the KEGG Orthology-based Annotation System software (http://kobas.cbi.pku.edu.cn/). Functional terms and pathways for DEGs with a corrected P ≤ 0.05 were considered significantly enriched. WEGO software was then used to obtain GO annotations and functional classifications of novel transcripts. The free online platform OmicShare Tools was used for data analysis (www.omicshare.com/tools). Data were displayed using SigmaPlot v.11.

### qRT-PCR

To verify the RNA-seq results, nine genes were selected for qRT-PCR analysis using *GADPH* as the internal reference gene^32^. Reactions were performed in a LightCycler 480 system (Roche, Basel, Switzerland) with 5 μL SYBR Premix Ex Taq II (Tli RNaseH Plus) (Takara Bio, Dalian, China), 1.5 μL cDNA template, 0.4 μL each primer (10 μmol/μL), and 2.7 μL nuclease-free water. Each reaction was prepared in triplicate. Denaturation took place at 95 °C for 30 s, followed by 40 amplification cycles of 95 °C for 5 s and 60 °C for 30 s. Melting curve analyses were performed at the end of the 40 cycles (95 °C for 5 s followed by a constant increase from 60 °C to 95 °C). Relative gene-expression levels were normalized to that of *GADPH* and determined with the 2^−ΔΔCT^ method^31^. SigmaPlot v.11 software was used for statistical analysis and to display the data.

### Data availability

All sequence data were deposited in the NCBI Sequence Read Archive (SRA, http://www.ncbi.nlm.nih.gov/Traces/sra) under accession numbers SRX2902847, SRX2913033, SRX2913042, SRX2913055, SRX2913085, and SRX2913357 for S1-1, S1-2, S3-1, S3-2, S5-1, and S5-2, respectively.

## Electronic supplementary material

Supplementary Information

Supplementary Table S1-S5
