# Supplementary material for: Transcriptomic analysis of the regulation of stalk development in flowering Chinese cabbage (Brassica campestris) by RNA sequencing
Source: Sci Rep. 2017 Nov 14;7:15517. doi: 10.1038/s41598-017-15699-6 (PMC5686075; doi:10.1038/s41598-017-15699-6)
Supplement: Supplementary file 1 — Supplementary Information [file 41598_2017_15699_MOESM1_ESM.pdf]

Supplementary Information

Transcriptomic analysis of the regulation of stalk development in flowering Chinese cabbage (*Brassica campestris*) by RNA sequencing

Xinmin Huang, Yuling Lei, Hongling Guan, Yanwei Hao, Houcheng Liu, Guangwen Sun, Riyuan Chen*, and Shiwei Song*

Guangdong Provincial Engineering Technology Research Center for Protected Horticulture, College of Horticulture, South China Agricultural University, Guangzhou 510642, Guangdong, China

* The corresponding authors, R.C. (email: rychen@scau.edu.cn) and S.S. (email: swsong@scau.edu.cn) contributed equally to this work.

Supplementary Figure

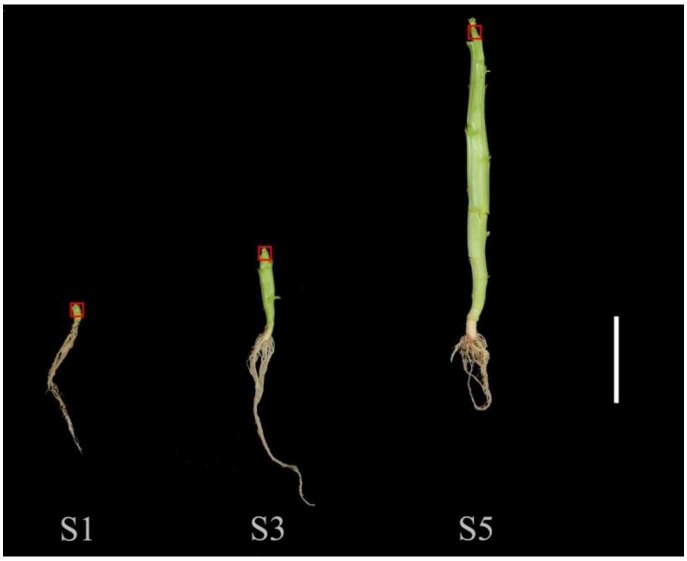

Figure S1. Changes in stalks during development in three stages, namely the seedling stage (S1), bolting stage (S3), and flowering stage (S5). The red box shows the sampling section used for RNA-seq and anatomical analysis. Bar = 5 cm.

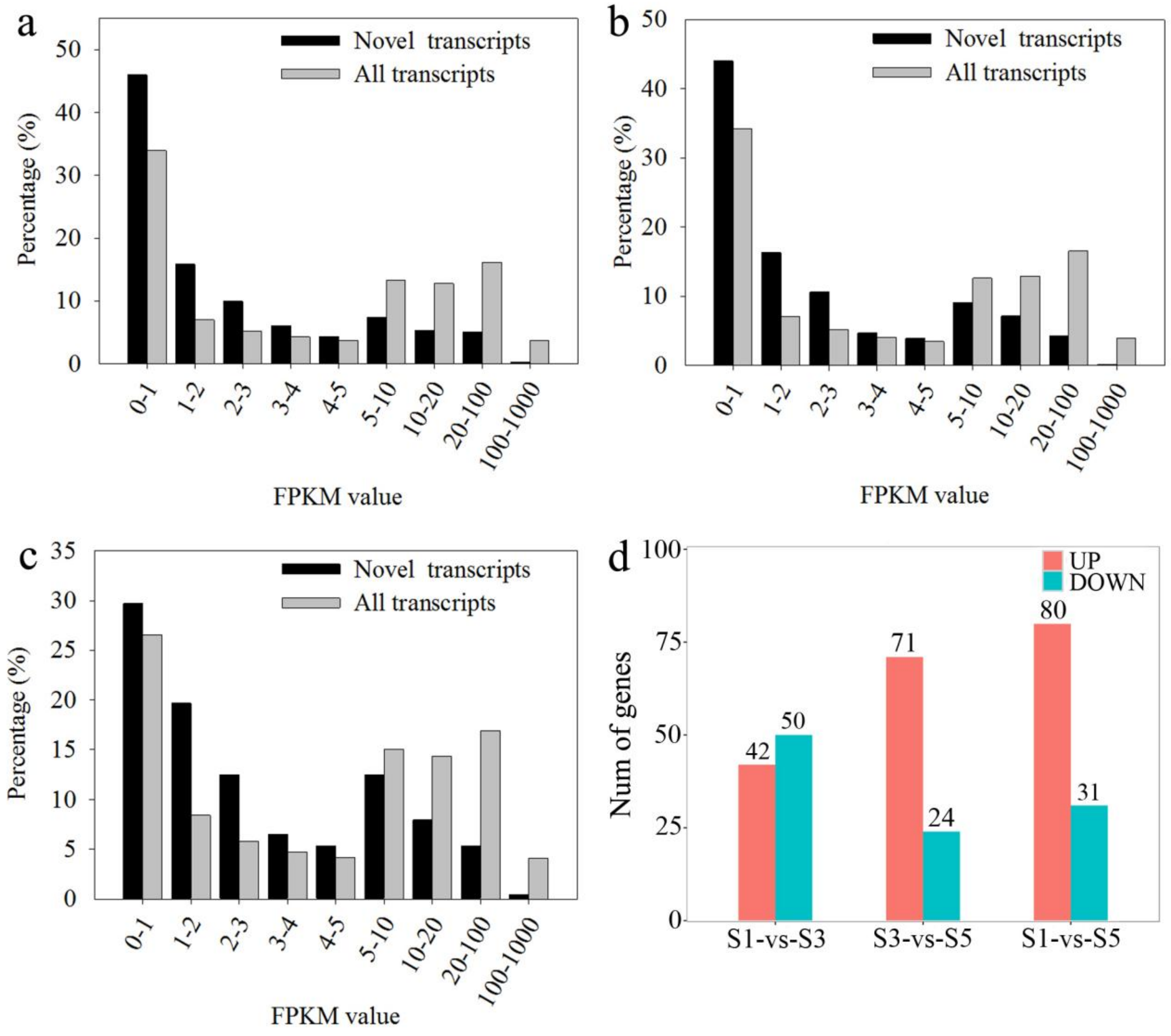

Figure S2. Expression pattern of novel transcripts. (a) Stage S1. (b) Stage S3. (c) Stage S5. (d) Pairwise comparisons of novel transcript expression levels. The numbers of up- and down-regulated genes in each pair of stages is presented.

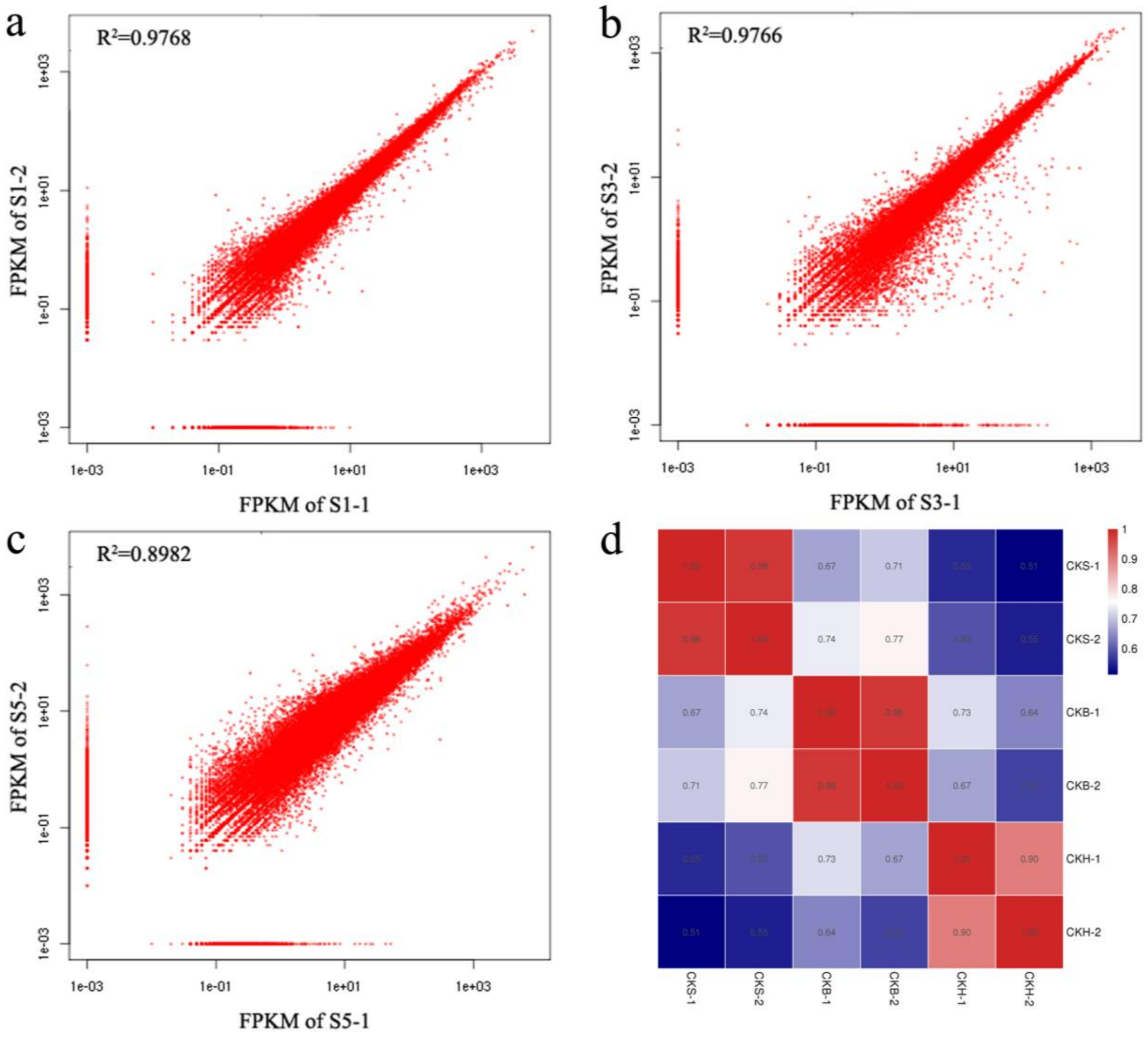

Figure S3. Correlation between gene-expression levels in two biological replicates for each stage. Pearson's correlation coefficients between (a) S1-1 vs. S1-2, (b) S3-1 vs. S3-2, (c) S5-1 vs. S5-2, and (d) six samples. $R^2 > 0.8$ was used as the cut-off for statistical significance.

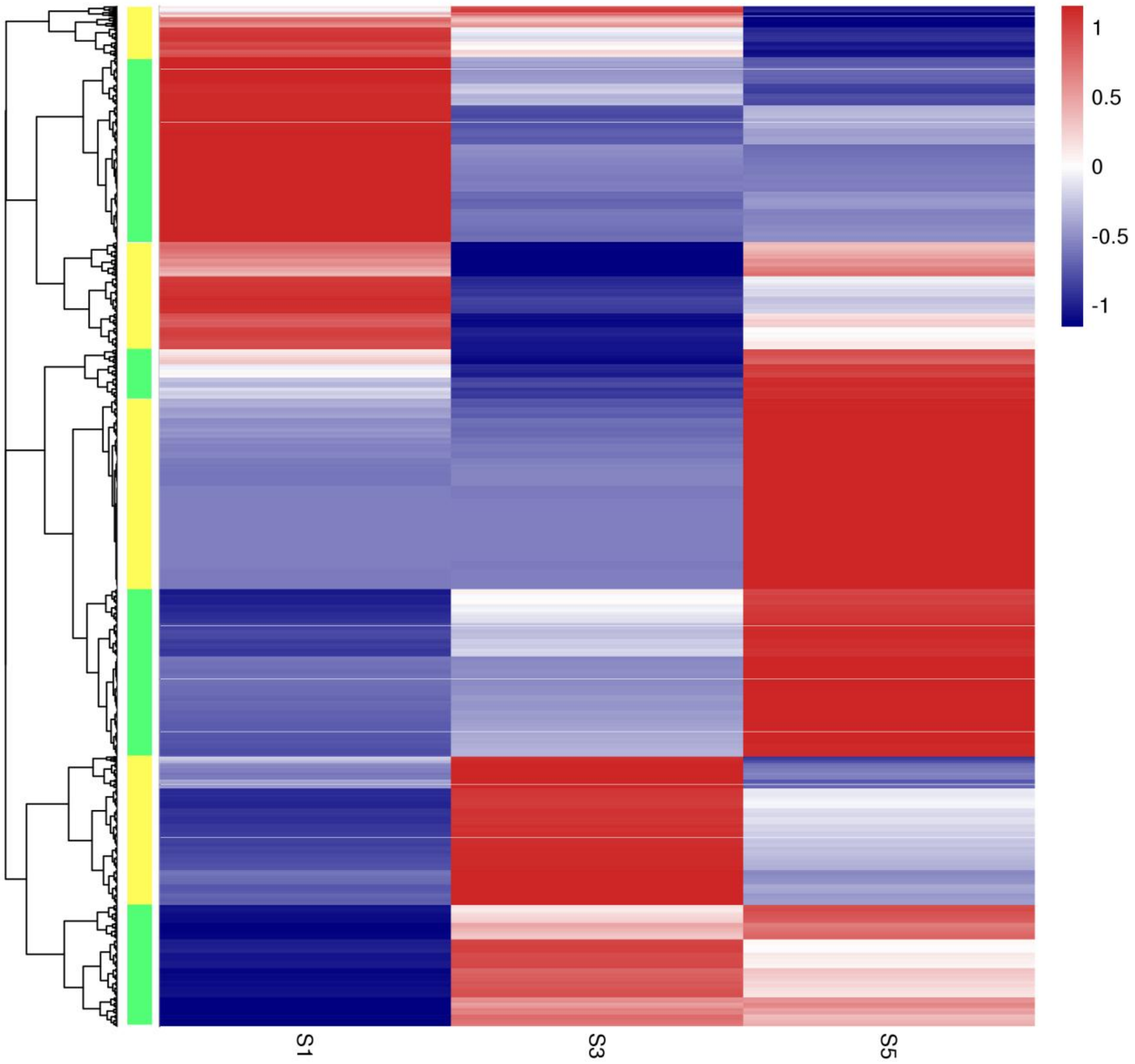

Figure S4. Heatmap of DEGs across three stalk-development stages in the flowering Chinese cabbage. Expression values of three stages presented as FPKM values normalized with the row Z-score. Eight main clusters are shown by the yellow and green bars, which were clustered using the Euclidean distance method associated with complete-linkage.

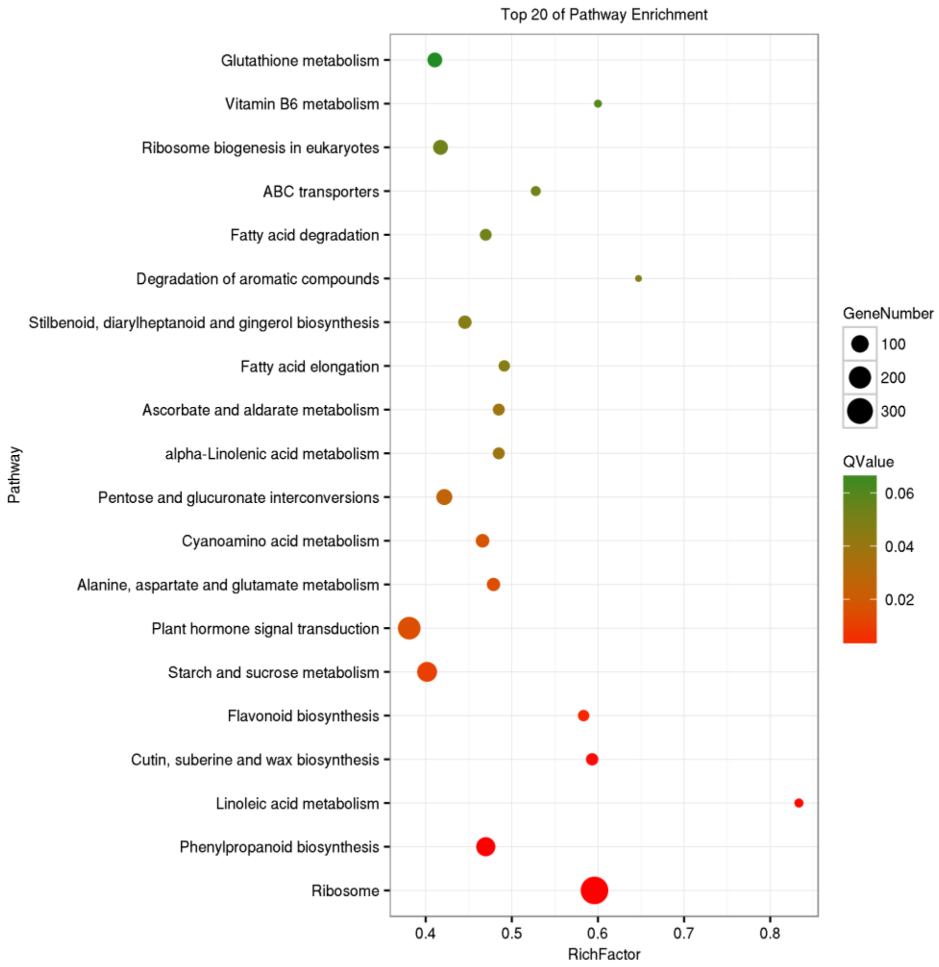

Figure S5. Top twenty pathway enrichments in all DEGs.

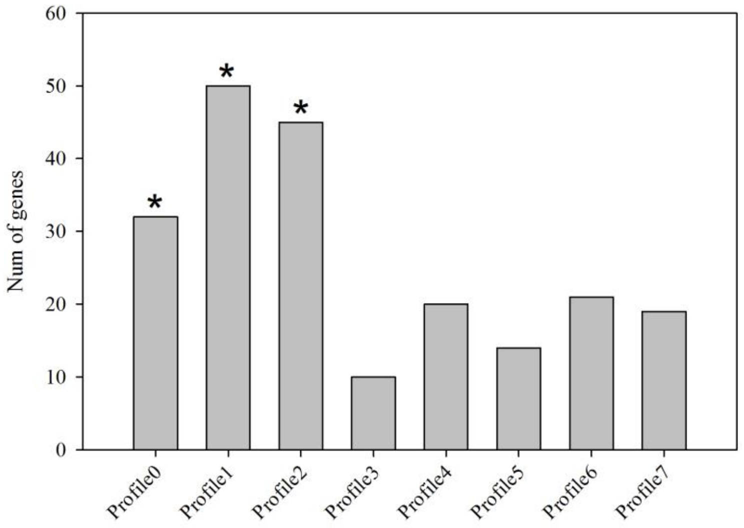

Figure S6. Number of genes in plant hormone signal-transduction pathways in each profile. “*” indicates a plant hormone signal-transduction pathway that was significantly enriched in a gene-expression profile (q value < 0.05).

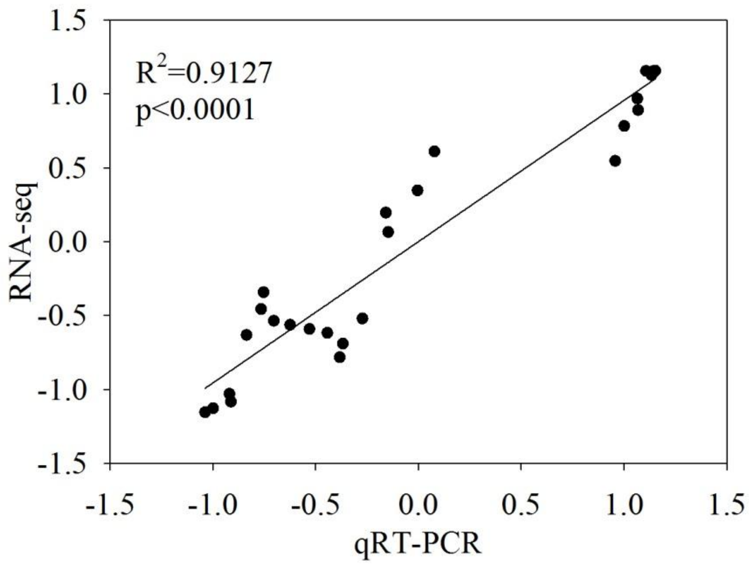

Figure S7. Validation of the RNA-seq gene-expression data by qRT-PCR. Nine DEGs from the RNA-seq data were used for qRT-PCR assays. Pearson's correlation coefficients between the RNA-seq and qRT-PCR data were unexceptionable, with $R^2 > 0.8$ as the significance threshold. The row Z-scores of the FPKM and qRT-PCR data are shown.

Supplementary Table

Table S1. List of novel transcripts that were annotated.

Table S2. GO terms (q value < 0.05) significantly enriched in the biological process category by DEGs in flowering Chinese cabbage.

Table S3. Top ten biological GO process significantly enriched in profiles 1, 4, 6, and 7.

Table S4. Flowering time-associated genes in flowering Chinese cabbage.

Table S5. Gene information and sequences of primers used for qRT-PCR.
